# Synthetic photorespiratory bypass more stably increases potato yield per plant by improving photosynthesis

**DOI:** 10.1111/pbi.70076

**Published:** 2025-04-02

**Authors:** Xiuling Lin, Yuming Long, Zhen Yao, Boran Shen, Min Lin, Xiaofen Zhong, Xiaohong Chen, Xiangyang Li, Guohui Zhu, Zhisheng Zhang, Xinxiang Peng

**Affiliations:** ^1^ State Key Laboratory for Conservation and Utilization of Subtropical Agro‐Bioresources, College of Life Sciences South China Agricultural University Guangzhou China; ^2^ College of Horticulture and Food, Guangdong Eco‐Engineering Polytechnic Guangzhou China; ^3^ College of Horticulture and Gardening Yangtze University Jingzhou China

**Keywords:** photorespiratory bypass, photosynthesis, yield, potato

## Abstract

The bioengineering of photorespiration has emerged as a key target for improving photosynthesis and crop yield. In our previous study, two photorespiratory bypasses, GOC and GCGT, were successfully established in rice, and the transgenic plants exhibited increased photosynthesis and yield. However, reduced seed‐setting rates were observed in both GOC and GCGT rice. To overcome this bottleneck, we introduced the GOC bypass into potato, as potato is vegetatively reproduced without the need for pollination, unlike rice. After the GOC bypass was successfully established in potato, transgenic plants were tested in field experiments at different locations in China with contrasting climates. Consequently, the yield per plant increased by 21.3%–69.2% for GOC potatoes under normal growth conditions and enhanced by 12.9%–29.9% under adverse environments. GOC potatoes acquired a more stable yield increase than GOC rice. Moreover, the advantages under high light, as noticed earlier for GOC rice, were further verified in this study through various field experiments because the yield increase was obviously higher in GOC potatoes grown in the northern area with high solar radiation than in those grown in the south with relatively lower solar radiation. Mechanistic analyses indicated that photosynthesis increased while photorespiration was suppressed, and much fewer photosynthates accumulated in GOC potatoes. These results demonstrate that the GOC bypass increases yield per plant more stably in potato than in rice, as well as show promising prospects for practical application in improving crop yields, particularly under high‐light conditions.

## Introduction

Increasing crop yields is the most practical approach to meeting the anticipated doubling of global food demand by 2050 (Long *et al*., 2015; Ray *et al*., 2013; South *et al*., 2019; Tilman *et al*., 2011). The yield strongly depends on an increase in biomass, which is primarily associated with photosynthesis (Croce *et al*., 2024; Heyneke and Fernie, 2018). However, the photosynthetic conversion efficiency is currently only 1%–2%, which is approximately one‐fifth of the theoretical value (Long *et al*., 2015; Zhu *et al*., 2010). This implies that the notable potential for photosynthetic efficiency remains to be explored (Long *et al*., 2015). Therefore, predictably, “a second Green Revolution” may occur in this century owing to improved photosynthesis (Long *et al*., 2006, 2015; Zhu *et al*., 2008). Photorespiration is a light‐dependent process in which O_2_ is absorbed and CO_2_ is released simultaneously (Bauwe *et al*., 2010; Betti *et al*., 2016). It metabolizes the product of the ribulose‐1,5‐biphosphate oxygenation reaction, thereby reducing the efficiency of photosynthesis by removing carbon from the Calvin cycle and consuming NADPH and ATP (Maurino and Peterhansel, 2010; Peterhansel and Maurino, 2011). Abiotic stresses such as drought, heat, and high light may stimulate photorespiration, making it even more wasteful. Therefore, photorespiration is often considered a process that significantly lowers the overall photosynthetic efficiency (Wingler *et al*., 2000). Modelling predicted that without photorespiration, photosynthesis would increase by 12%–55% (Eisenhut *et al*., 2019; South *et al*., 2019; Walker *et al*., 2016b). Therefore, photorespiration is a key limiting factor to be optimized for improving photosynthesis and subsequently increasing crop yields (Peterhansel *et al*., 2008; South *et al*., 2018; Xin *et al*., 2015; Zhu *et al*., 2008).

The introduction of photorespiration bypasses has been investigated in different plants (Fernie and Bauwe, 2020; Jin *et al*., 2023; Smith *et al*., 2023). Each bypass aimed to use photorespiratory CO_2_ to form a photosynthetic CO_2_ concentrating mechanism, thereby reducing the loss of photorespiration. In the first bypass (GGT), three *Escherichia coli* enzymes, glycolate dehydrogenase (EcGDH), glyoxylate carboligase (EcGCL), and tartronic semialdehyde reductase (EcTSR), were introduced into Arabidopsis chloroplasts (Kebeish *et al*., 2007). This strategy was subsequently adopted by Dalal *et al*. (2015) for *Camelina sativa*; Nölke *et al*. (2014) and Ahmad *et al*. (2016) for potato; Chen *et al*. (2019) for cucumber; South *et al*. (2019) for tobacco; and Wang *et al*. (2020) and Nayak *et al*. (2022) for rice. Almost all these studies showed that GGT bypass increased photosynthesis, biomass, and even grain yields. The second bypass (GMC), where glycolate was completely oxidized to CO_2_ in the chloroplasts of Arabidopsis by introducing glycolate oxidase, malate synthase, and catalase, also enhanced photosynthesis and biomass (Maier *et al*., 2012). Its performance was further verified recently both in tobacco by South *et al*. (2019) and in rice by Xu *et al*. (2023).

We have previously reported a distinct bypass called GOC, which comprises three enzymes from rice: glycolate oxidase (OsGLO3), oxalate oxidase (OsOxO3), and catalase (OsCATC). The transgenic rice plants exhibited significant increases in photosynthetic efficiency, biomass, and grain yield under greenhouse and field conditions (Shen *et al*., 2019). However, their grain yield is unstable and fluctuates in different cultivation seasons because of reduced seed‐setting rates (Shen *et al*., 2019). To address this limitation, bioengineering the bypass into tuberous crops may be a more feasible method because tuber yield is vegetatively reproduced without necessitating pollination, which is required for rice. Potato (*Solanum tuberosum* L) is crucial for global food security (Ahmadu *et al*., 2021; Devaux *et al*., 2014, 2020) and a vital food commodity (Devaux *et al*., 2014; Krizkovska *et al*., 2022; Zhang *et al*., 2017). It may be a substitute for grain crops in the future because of its high nutritional value, adaptability to diverse environments, and potential yield (Azimuddin *et al*., 2009; Burgos *et al*., 2020; Zhang *et al*., 2017). Therefore, in this study, potato was selected to have an engineered GOC bypass. After the bypass was successfully established in potato, its performance was tested through various field experiments conducted at different locations in China. The results showed that the yield per plant of GOC potato was more stably increased via improving photosynthesis, as compared to that of GOC rice. Increases in yield per plant were observed even under adverse environmental conditions. More notably, GOC potato also showed strong light advantages, similar to GOC rice. This study demonstrates that GOC bypass increases yield per plant more stably in potato than in rice, but also shows promising prospects for practical application in improving crop yields, particularly under high‐light conditions.

## Results

### Construction of GOC potato

**Figure 1:**
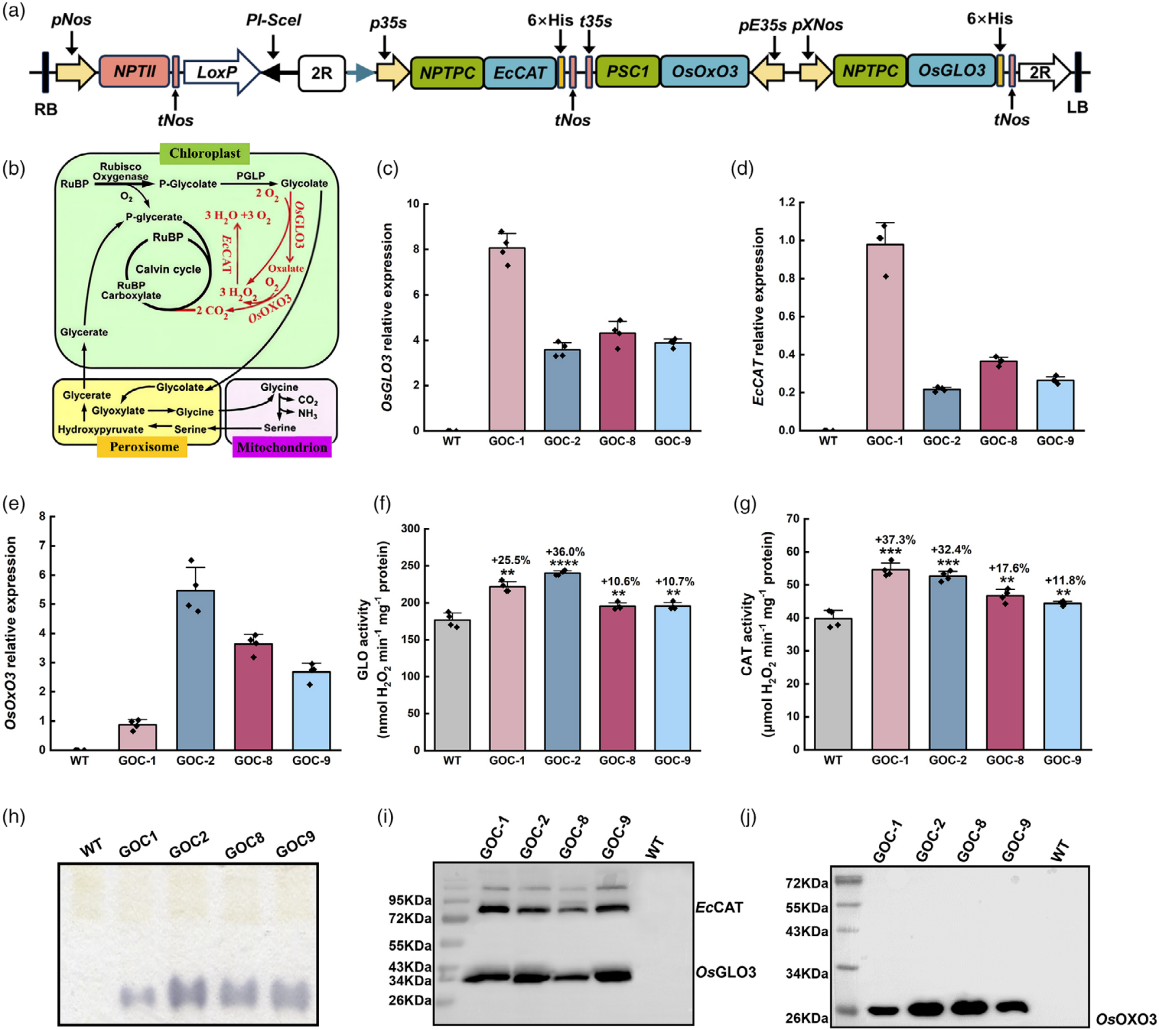
Establishment of the GOC photorespiratory bypass in potato plants. (a) Structure of the multigene expression vector GOC*‐pBIA13*. The core region of vector *pBIA13* is detailed in Figure S1; *pNos*, *Nos* promoter; *tNos*, *Nos* terminator; *p35S*, *CaMV35S* promoter; *pE35S*, *CaMV 35S* enhanced promoter; *t35S*, *CaMV35S* terminator; *pXNos*, prolonged *Nos* promoter; *NPTII*, expression cassette of neomycin phosphotransferase gene; *NPTPC* and *PCS1*, potato rbcS chloroplast localization signal peptide; *OsGLO3*, rice glycolate oxidase 3; *EcCAT*, *Escherichia coli* catalase; *OsOxO3*, rice oxalate oxidase3; LB, left border; RB, right border. (b) The modified GOC bypass (in red), including the native photorespiration pathway (in black). Rubisco, ribulose‐1,5‐bisphosphate carboxylase/oxygenase; PGLP, phosphoglycolate phosphatase. (c–e) mRNA transcript abundances of *OsGLO3*, *EcCAT*, and *OsOxO3* in GOC plants. Total RNA extracted from the leaves of wild‐type (WT) and GOC potato plants was used for real‐time RT‐qPCR analysis with gene‐specific primers as listed in Table S4; the potato *APT* (*CK270447*) gene served as the endogenous control. (f, g) Activities of GLO and CAT in GOC potato plants. (h) Detection of OxO activities using the CN‐page method. (i, j) Expression patterns of *Os*GLO3, *Ec*CAT, and *Os*OxO3 in GOC potato plants. Total protein extracted from leaves of WT and GOC potato was analysed by western blot. *Os*GLO3 and *Ec*CAT were detected by the anti‐His tag monoclonal antibody, and *Os*OxO3 was detected by the anti‐OxO polyclonal antibody. Data are presented as the mean ± SD, *n =* 4; **P < 0.01, ***P < 0.001, ****P < 0.0001 according to Student's *t*‐test.

The GOC bypass, previously designed for rice by Shen *et al*. (2019), was modified to fit dicotyledonous potato. The key modifications included changes in promoters and signal peptides (Figures S1 and S2). After the multigene vector GOC‐*pBIA13* was assembled (Figure 1a), it was transformed into potato via *Agrobacterium*‐mediated transfection. Four representative lines were selected from the number of positive independent lines for further analysis. Molecular identification showed that the three target genes were well expressed at the mRNA (Figure 1c–e), protein (Figure 1i,j), and activity levels (Figure 1f–h), indicating that the GOC bypass was successfully established in potato plants.

### Yield and biomass of GOC potato

To test the GOC potato plants generated, field experiments were conducted for years at different locations in China, ranging from the south to the north across latitudes from 23°07′53″ to 40°50′25″ N.

#### Field trials in Guangzhou

**Figure 2:**
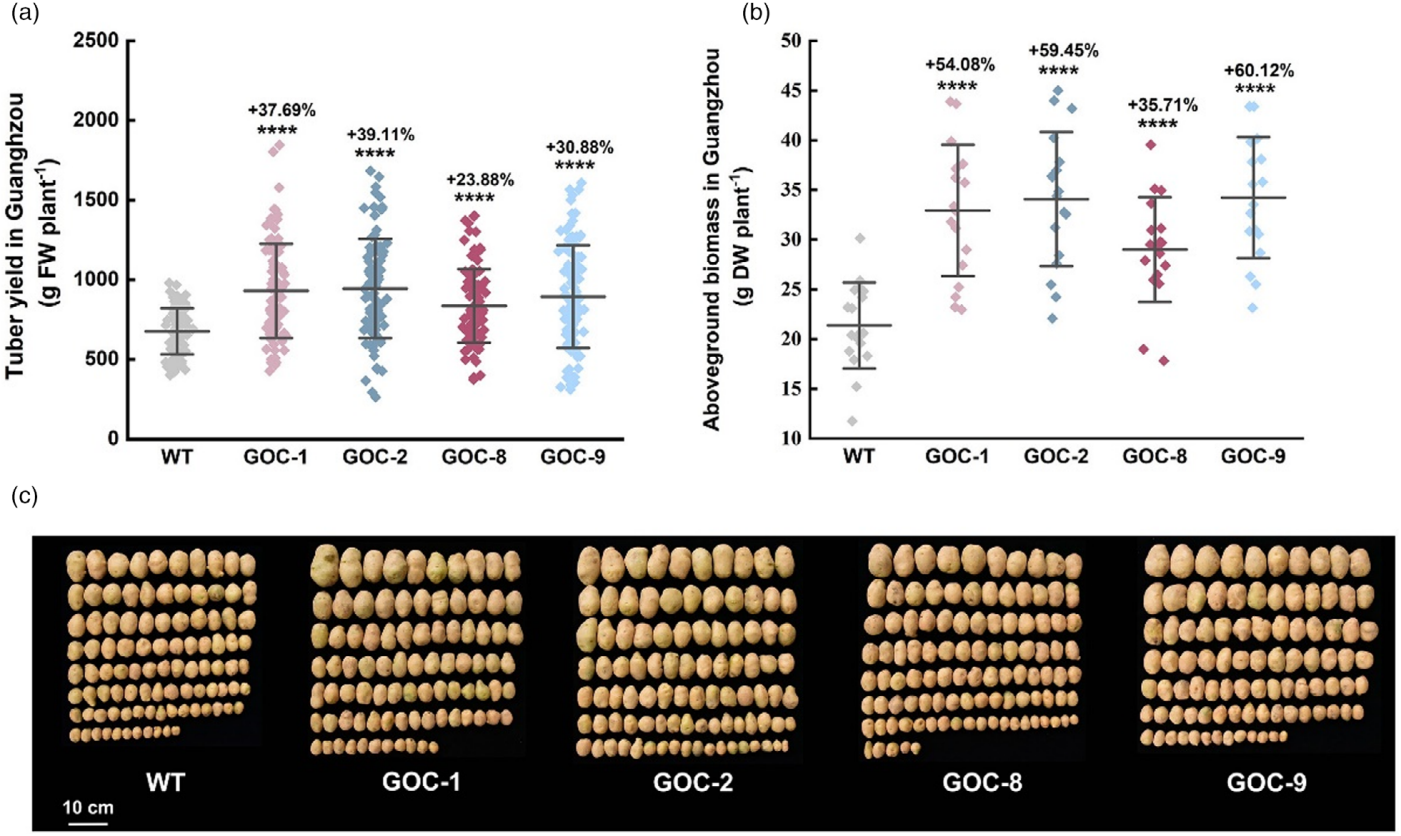
Yield and biomass of GOC potato planted in Guangzhou in 2024. (a) Yield per plant of GOC potato planted in Guangzhou, *n* = 97. (b) Biomass of GOC potato planted in Guangzhou, *n =* 18. (c) Photos of tubers harvested from 10 potato plants of WT and GOC grown in Guangzhou. Data are presented as the mean ± SD; ****P < 0.0001 according to Student's *t*‐test.

In Guangzhou, South China, the experiments were conducted for years, and the data from 2024 were adopted and shown in Figure 2, where four independent GOC lines (GOC1, 2, 8, and 9) were tested. The yield per plant of these GOC potato plants increased by 23.9%–39.1% (Figure 2a,c), and the aboveground biomass increased by 35.7%–60.1%, compared with wild‐type (WT) plants (Figure 2b). The experiments in 2020 and 2023 further demonstrated significant increases in yield per plant of GOC potato, ranging from 25.5%–37.4% (Figure S3).

#### Field trials in other regions of China

GOC potato plants were grown in different regions of China to confirm the above results. In Hunan, south‐central China, the yield per plant increased by 21.3%–30.5% (Figure S4a), and the aboveground biomass increased by 16.0%–18.7% (Figure S4b). In northwest China, Gansu, the yield per plant increased by 31.8%–69.2% (Figure S4c), and the aboveground biomass increased by 19.9%–56.9% (Figure S4d). In northern Inner Mongolia, the yield per plant increased by 31.2%–47.0% (Figure S4e), and the aboveground biomass increased by 6.5%–19.9% (Figure S4f).

#### Field trials under adverse conditions

When GOC potato plants were grown in Tianjin from mid‐April to mid‐July 2021, long rainy days with storms were encountered (July 2021), and the plants were subjected to intermittent waterlogging for approximately 10 days at the late stage. Even under such conditions, the yield per plant of GOC potato plants increased to approximately 20% higher than that of the WT (Figure S5a). When GOC potatoes were planted in Hunan from early February to mid‐May 2024, even longer rainy days were experienced (April–May 2024), but the plants were not flooded. Under such long, low light, and rainy conditions, the yield per plant increased by 12.9%–29.9% (Figure S5b).

Collectively, these results indicate that GOC bypass can consistently increase the yield of potatoes, even under adverse conditions.

### Photosynthesis in GOC potato

**Figure 3:**
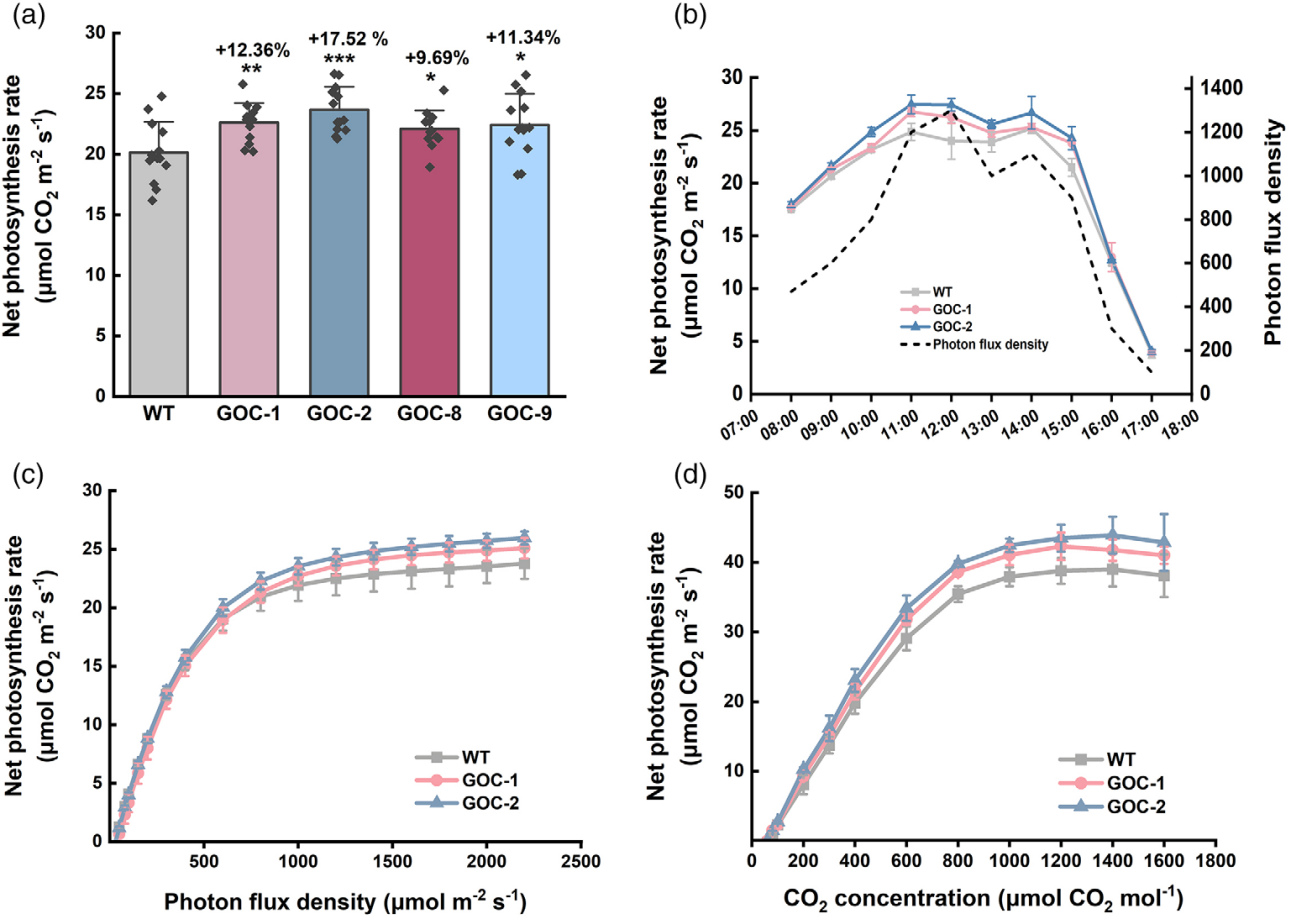
Photosynthesis in GOC potato. (a) Net photosynthetic rates, *n =* 13. (b) The diurnal curves of net photosynthetic rate, *n =* 6. (c) Light‐response curves, *n =* 6. (d) CO_2_‐response curves, *n =* 6. Plants were planted in Guangzhou. All measurements were conducted using the fourth fully expanded leaf from the top of the potato at the tuber swelling stage. Data are presented as the mean ± SD; *P < 0.05, **P < 0.01, ***P < 0.001 according to Student's *t*‐test.

We measured gas exchange in the GOC and WT plants. The photosynthetic rate (Pn) was 9.7%–17.5% higher (Figure 3a), with GOC potato plants exhibiting an advantageous diurnal Pn for most of the daytime (Figure 3b). To confirm the enhanced photosynthesis, light‐ and CO_2_‐response curves were further analysed (Figure 3c,d). GOC plants showed higher Pn when the photon flux density (PFD) was over 400 μmol m^−2^ s^−1^, and the maximum difference occurred at approximately 2000 μmol m^−2^ s^−1^ (Figure 3c). A higher Pn was also observed in GOC plants under different intracellular CO_2_ concentrations (Figure 3d). In addition, the key quantitative traits defining the light‐response and CO_2_‐response curves were estimated by Ye *et al*. (2013) using the online Photosynthesis Model Simulation Software (PMSS) (http://photosynthetic.sinaapp.com/) (Table S1). The results showed that the maximum carboxylation efficiency (*V*_cmax_), light saturation point (LSP), and light‐saturated photosynthetic rate (*A*_max_) were all significantly increased in GOC plants (Table S1). These results collectively indicate that photosynthetic performance is improved in GOC potato plants.

**Figure 4:**
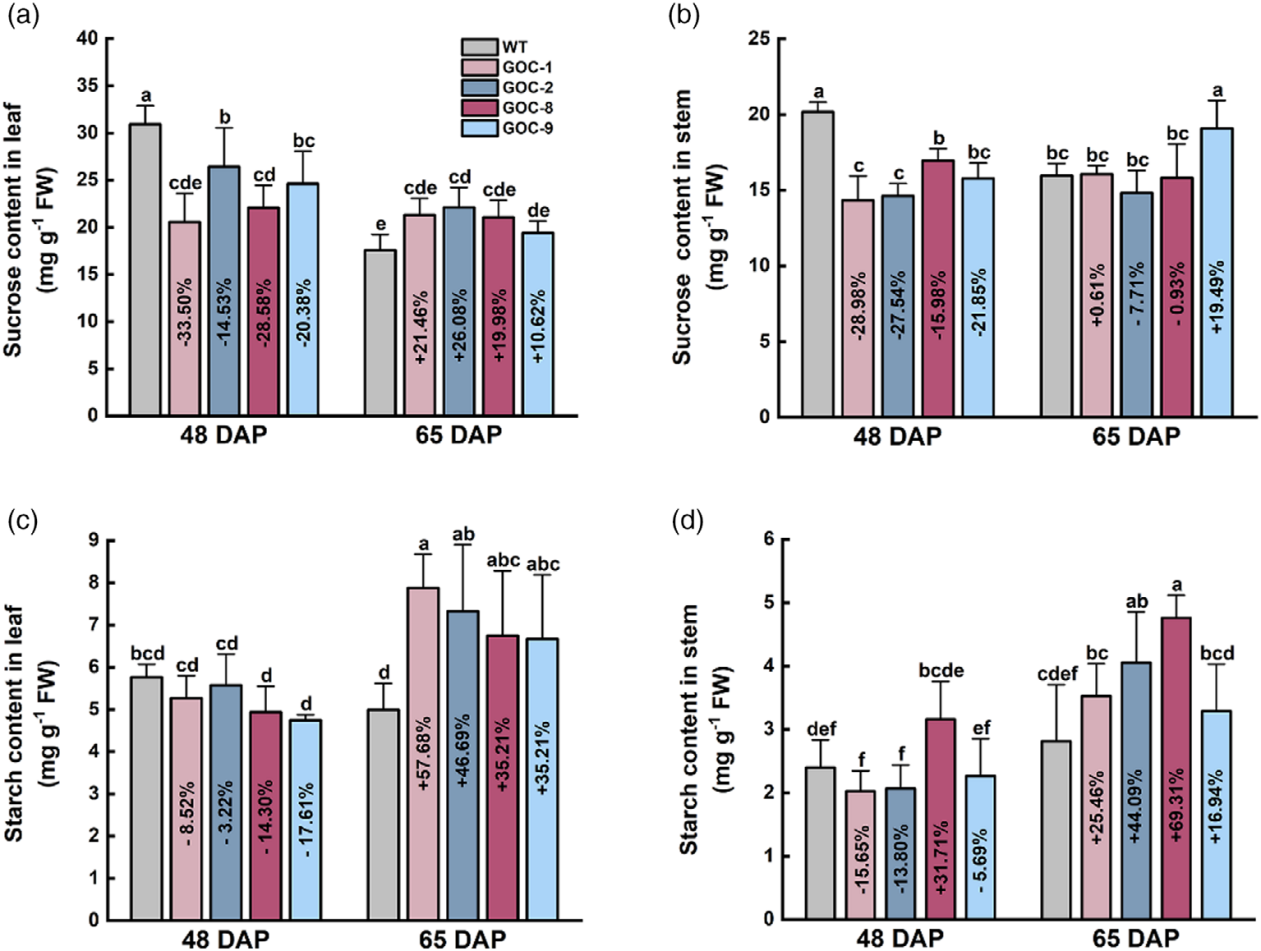
Photosynthates in GOC potato. (a) Sucrose content in leaves. (b) Sucrose content in stems. (c) Starch content in leaves. (d) Starch content in stems. WT and GOC potatoes grown in Guangzhou were sampled 48 and 65 days after planting (DAP). Data are presented as the mean ± SD; different letters indicate significant differences (*n =* 4, *P* < 0.05, Duncan's test).

Photosynthates are typically used as cumulative proxies to evaluate photosynthesis. As shown in Figure 4, sucrose was 14.5%–33.5% and 16.0%–29.0% lower in the leaves and stems, respectively, of GOC potato plants at 48 DAP, but no such differences were observed at 65 DAP (Figure 4a,b). Starch was not significantly different between GOC and WT plants at 48 DAP, while it increased by 35.2%–57.7% and 16.9%–69.3% in the leaves and stems of GOC plants at 65 DAP, respectively (Figure 4c,d). These results indicate that in GOC potato, sucrose does not accumulate, and starch accumulates only at the late stage, in contrast to GOC rice (Shen *et al*., 2019).

### Photorespiration in GOC potato

**Figure 5:**
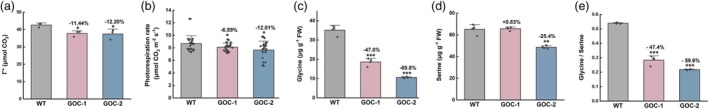
Photorespiration in GOC potato. (a) Γ* (photorespiratory CO_2_ compensation point) measured using the Laĭsk method, *n =* 5. (b) Photorespiration rates measured using the low O_2_ method, *n =* 30. (c, d) Leaf glycine and serine levels. (e) Leaf glycine/serine ratio. Metabolite contents relative to the internal standard ribitol were measured by gas chromatography–mass spectrometry using leaf samples harvested between 14:00 and 16:00, *n =* 4. Data are presented as the mean ± SD; *P < 0.05, **P < 0.01, ***P < 0.001 according to Student's *t*‐test.

The GOC bypass may mechanistically suppress photorespiration by increasing CO_2_ concentrations inside chloroplasts. The photorespiratory CO_2_ compensation point (Γ*) is commonly used to indicate photorespiration (Walker *et al*., 2016a). As shown in Figure 5a, Γ* was significantly lower in GOC potato plants than in the WT plants, indicating suppressed photorespiration in GOC potato plants. To further verify the result, photorespiratory rates were directly determined using the low oxygen method (Wujeska‐Klause *et al*., 2019). The results showed that the photorespiratory rate was reduced by 6.6%–12.0% in GOC potato plants (*P* < 0.05) (Figure 5b). In addition, amino acids were also determined in potato leaves (Table S2) because glycine and serine levels have been regarded as indicators of carbon flux through photorespiration, and glycine/serine ratios are positively correlated with photorespiration (Fu *et al*., 2023; Kebeish *et al*., 2007; Novitskaya *et al*., 2002; Sousa *et al*., 2023). As shown in Table S2 and Figure 5c–e, glycine levels were considerably reduced by 47.0%–69.8% in GOC potatoes, such that the glycine/serine ratio was 47.4%–59.6% lower than WT. These results support that photorespiration is reduced in GOC potatoes.

## Discussion

Biomass is essential for yield formation and depends primarily on photosynthesis (Heyneke and Fernie, 2018; Liu *et al*., 2022; Wu *et al*., 2019; Yamori, 2021). Previous experiments on fertilizer application have observed a positive correlation between biomass and yield in various crops such as rice (Khairunniza‐Bejo *et al*., 2016), cotton (Luo *et al*., 2020; Shi *et al*., 2022), barley (Agegnehu *et al*., 2012), corn (Biswas and Ma, 2016), banana (Zhang *et al*., 2020), and faba bean (Agegnehu *et al*., 2005; Agegnehu and Fessehaie, 2006; Weldua *et al*., 2012). Recent transgenic studies have further demonstrated that biomass directly affects crop yields (Yamori, 2021). For example, transgenic plants, including rice, wheat, cucumber, and *Camelina sativa*, exhibited increased biomass and seed yields (Chen *et al*., 2019; Dalal *et al*., 2015; Driever *et al*., 2017; Kumar *et al*., 2009; Kurek *et al*., 2007; Nayak *et al*., 2022; Rosenthal *et al*., 2011; Scafaro *et al*., 2010). In contrast, evidence showed that yield is dependent on biomass as well as the coordination between source and sink flow. In this case, biomass does not necessarily correlate with yield, and a high biomass variety may result in a poor yield. For example, some wild rice relatives accumulate high biomass but have poor grain yields (Sanchez *et al*., 2013).

To increase photosynthesis, suppressing photorespiration is considered an effective way (Eisenhut *et al*., 2019). There have been different strategies aiming at reducing photorespiration (Fernie and Bauwe, 2020; Garcia *et al*., 2023; Jin *et al*., 2023; Smith *et al*., 2023; Zhang *et al*., 2024). Engineering Rubisco has long been a key target since its additional oxygenase activity leads to photorespiration. By employing protein engineering and molecular breeding techniques to optimize Rubisco, its CO_2_ affinity or specificity was reportedly increased, such that photorespiration was reduced. Scientists are also investing in introducing non‐plant CO_2_ concentrating mechanisms (CCMs) into C_3_ plants, which have evolved in algae and cyanobacteria. This engineering is expected to increase CO_2_ concentration around Rubisco, thereby suppressing photorespiration. In addition, introducing C_4_ plant photosynthetic pathways into C_3_ plants is currently a hot research field. This involves expressing key C_4_ enzymes and attempting to reconstruct leaf structures similar to those of C_4_ plants, conferring low photorespiration in C_3_ plants like C_4_ plant. Lastly, alternative photorespiratory pathways have been demonstrated to be another efficient way, which metabolize 2‐phosphoglycolate (2‐PG) directly within chloroplasts to increase chloroplastic CO_2_ concentrations and thus suppress photorespiration. More significantly, among the above strategies, photorespiratory bypasses are the only ones shown to enhance photosynthesis and productivity, even grain yield in the field (Garcia *et al*., 2023; Zhang *et al*., 2024).

In our previous study, two photorespiratory bypasses, GOC and GCGT, were successfully established in rice plants, exhibiting increased photosynthesis, biomass, and grain yield. However, a negative phenotype, that is, reduced seed‐setting rates, was observed in both types of bypass rice, such that their yield increases were not consistent, with a 7%–27% increase in spring and a 13%–16% decrease in the fall (Shen *et al*., 2019; Wang *et al*., 2020). Our recent mechanistic study revealed that in GCGT rice, sugar accumulation served as the primary signal that resulted in a reduced seed‐setting rate, during which impaired pollen fertility occurred owing to a metabolic disorder of sugar in the anthers (Li *et al*., 2024). Therefore, we speculated that the limitation encountered in rice might be resolved or improved by introducing the bypass into tuberous crops, as they are vegetatively reproduced without pollination as required for rice. Potato was selected based on its agricultural importance and technical feasibility. Subsequently, the GOC bypass was successfully introduced into the potato after the multigene vector was modified to fit the dicot potato. The GOC bypass functionally increased photosynthesis in rice by suppressing photorespiration (Shen *et al*., 2019). In this study, GOC potato showed similar results, such as significant increases in Pn (Figure 3), LSP (Table S1), *A*_max_, and *V*_cmax_ (Table S1), and intercellular CO_2_ concentrations (Figure S7), with reductions in Γ* (Figure 5a), photorespiratory rates (Figure 5b) and glycine/serine ratios (Figure 5e). More importantly, various field experiments at different locations in China, even under adverse environments, suggested that the GOC bypass is relatively more consistent for increasing yield in potatoes (Figure 2 and Figures S3 and S4), in contrast to GOC rice (Shen *et al*., 2019).

A moderate increase in photosynthesis (9.7%–17.5%) (Figure 3a) resulted in a high increase in yield per plant (21.3%–69.2%) in GOC potatoes (Figure 2 and Figures S3 and S4). Photosynthesis is a dynamic process that may vary over time, whereas yield is cumulative and represents the integrated carbon assimilation over the entire growing season; therefore, even a small increase in photosynthesis can translate into significant increases in biomass and yield (Heyneke and Fernie, 2018; Parry *et al*., 2011). Notably, relative to GOC rice, the GOC potato appeared to have a more efficient translation of photosynthates into yield, with a 9.7%–17.5% increase in Pn (Figure 3a), which was similar to that of GOC and GCGT rice (6%–23%) (Shen *et al*., 2019; Wang *et al*., 2020), and achieved a 21.3%–69.2% increase in yield per plant (Figure 2 and Figures S3 and S4), which tended to be higher than that for GOC and GCGT rice (7%–27%) (Shen *et al*., 2019; Wang *et al*., 2020). In addition, the yield increase was more consistent for GOC potatoes because consistent increases were observed when GOC potatoes were cultivated at different locations in China, even under adverse conditions (Figure S5), in contrast to GOC and GCGT rice, which showed decreased yields in the fall season, resulting from highly reduced setting rates (Shen *et al*., 2019; Wang *et al*., 2020). Photosynthate availability in source leaves is not the only factor that influences yield; long‐distance transport and sink strength also affect crop yield (Fernie *et al*., 2020; van den Herik *et al*., 2021). Thus, improvements in photosynthesis may have variable effects on crop yield. GOC potatoes acquired a more extensive source (increased photosynthesis, leaf area, and aboveground biomass) (Figure 3a; Figure S8; Figure 2b and Figures S4b, d, f) and an increased sink (increased tuber number) (Figure S9), similar to GOC and GCGT rice (Shen *et al*., 2019; Wang *et al*., 2020). However, in contrast to GOC and GCGT rice, which accumulated large amounts of sucrose and starch in the leaves and stems (Shen *et al*., 2019; Wang *et al*., 2020), no accumulation of sucrose was detected in the leaves and stems of GOC potato at different stages, and starch accumulated only at the late stage (Figure 4). Such a difference may ultimately result from the “flow” advantage of potato over rice.

Vascular bundles are the central transport system in plants that link sources to sinks (Claßen‐Bockhoff *et al*., 2021; Peterson *et al*., 1982). The size, structure, and capacity of vascular bundles affect the photosynthate transportation efficiency (Chang *et al*., 2022; Ren *et al*., 2021). Potatoes possess bicollateral vascular bundles that have phloem on the outer and inner sides of the xylem, and these bundles are organized as a ring throughout the plant tissues (Kaiser *et al*., 2006; Paul *et al*., 2017). In contrast, rice has closed‐type collateral vascular bundles with only outer phloem scattered throughout the plant tissues (Dzhamirze *et al*., 2021). Moreover, the inferior spikelet rachilla in rice cannot efficiently transport photosynthates because of poorly developed vascular bundles (You *et al*., 2021). Additionally, larger stems were observed in GOC potatoes, resulting from the development of secondary vascular tissues (Figure S10). In contrast, rice lacks the vascular cambium and thus is not capable of secondary growth, such that the stems of GOC and GCGT rice are not significantly different from those of the WT (Shen *et al*., 2019; Wang *et al*., 2020). All these results demonstrate that potato may have “flow” advantages owing to its superior capacity of vascular bundles over rice, typically with the extra‐developed secondary vascular tissues that can facilitate photosynthate transportation in plants (Turley and Etchells, 2022). In contrast to the vegetative reproduction of potato, pollination is required before rice grain filling. We recently detected that sugar accumulation acts as the primary signal, causing a reduced setting rate, during which impaired pollen fertility occurs owing to a metabolic disorder of sugar in the anthers (Li *et al*., 2024). Clearly, such an impact is avoided in potato, even if some photosynthate accumulation still occurred (Figure 4). Collectively, the “flow” advantage and asexual reproduction in potato may have contributed to the GOC bypass functioning more efficiently to potentially increase yield compared to rice.

GOC rice had a photosynthetic advantage under high‐light conditions, whereas other bypasses were reportedly advantageous only under low‐light and short‐day conditions (Kebeish *et al*., 2007; Maier *et al*., 2012; Peterhansel and Maurino, 2011). This study further observed that GOC potato also had high‐light advantages. Evidence initially emerged from the light response of photosynthesis, indicating that the maximum difference occurred at as high as 2000 μmol m^−2^ s^−1^ (Figure 3c), along with higher *A*_max_ and LSP values (Table S1). This advantage was further reinforced by the field experiments conducted at different locations under various conditions. GOC potatoes achieved more yield increases in areas with relatively higher solar radiation (Figure 2 and Figures S6 vs S11). For example, 31.2%–69.2% increases in yield per plant were observed in the northern areas (Figures S4c and S4e), where the total solar radiation was 888 370–915 218 W/m^2^ during the growth period (Figure S11 and Table S3). In contrast, 21.3%–39.1% increases in yield per plant occurred in the southern areas (Figure 2a and Figure S4a), where the total solar radiation was 462 969–616 871 W/m^2^ (Figure S11 and Table S3). Under high‐light intensity, ATP and NADPH produced via the light reaction are sufficient and even in excess; CO_2_ assimilation (dark reaction) becomes a limiting factor for photosynthesis; therefore, the photosynthetic CO_2_‐concentrating mechanism provided by the GOC bypass assumes a functional role. These results indicate that the GOC bypass increases potato yield under high‐light conditions.

In summary, the GOC photorespiratory bypass is more suitable to be bioengineered in tuberous crops, and the GOC potato has promising prospects for practical application in improving crop yields, particularly under high‐light conditions. More field tests with broader areas as well as with yield per block collected are underway, aiming to finalize its true extension and application value. More notably, the coordination between source‐flow‐sink is another key point deserving attention for us to increase crop yields via improving photosynthetic efficiency in the future.

## Experimental procedures

### Plasmid construction and generation of transgenic potato

The GOC*‐pBIA13* multigene expression vector was constructed as described by Shen *et al*. (2019) with minor modifications. The DNA sequence of the rbcS chloroplastic transit peptide (NPTPC) was amplified using PCR from the potato (*Solanum tuberosum*) *rbcS1* gene (*X69759.1*) (Yao *et al*., 2020). PCS1 is an extended transit peptide relative to *NPTPC*, with 16 amino acids from the cleavage site of the plastocyanin precursor protein peptide in *Silene pratensis* added to the C‐terminus of potato rbcS chloroplast localization peptide (StTP‐80AA) (Figure S2). The catalase‐coding sequence (*EcCAT*, *M55161.1*) was obtained from the *Escherichia coli* genome. The coding sequences of oxalate oxidase 3 (*OsOXO3*, *Os03g0693900*) and glycolate oxidase 3 (*OsGLO3*, *Os04g0623500*) were amplified from the total cDNA of WT rice (*Oryza sativa* L. ZH11). Six C‐terminal amino acids of *EcCAT* and *OsGLO3* were replaced with a 6 × His‐tag sequence. The *PCS1* and *NPTPC* sequences were then fused to the 5′‐terminus of the target genes by appropriate restriction enzyme sequences. The *NPTPC‐OsGLO3* and *NPTPC‐EcCAT* fusion genes were introduced into *pBIA13* under the control of prolonged *Nos* (agrobacterium carmine synthase) and *CaMV35S* promoters respectively, and *PCS1‐OsOxO3* fusion genes were introduced into *pBIA13* under the control of the *CaMV35S* enhanced promoter (Figure S2) (Yao *et al*., 2020). Further, these fusion gene cassettes were assembled into the multigene expression vector *pBIA13* using a Cre/loxP‐mediated site‐specific recombination method (Zhu *et al*., 2017). The primers used for the PCR are listed in Table S4. The constructed vector GOC*‐pBIA13* was introduced into potato via *Agrobacterium*‐mediated infection (strain *LBA4404*). The positive lines were identified by screening for kanamycin resistance. The WT potato (*S. tuberosum* cv. E‐potato 3) was kindly provided by the Potato Research Center at Huazhong Agricultural University, Wuhan, China.

### Growth conditions and yield evaluation

Field experiments were performed in five different locations of China, including Guangzhou (23°07′53″ N, 113°15′36″ E), south China; Huan (25°46′24″ N, 113°00′32″ E), south‐central China; Gansu (36°03′42″ N, 103°49′54″ E), northwest China; Tianjin (38°57′5″ N, 117°14′12″ E), and Inner Mongolia (40°50′25″ N, 111°44′35″ E), north China. The experiments were conducted in Guangzhou from mid‐November to mid‐March in 2020, 2023 and 2024; in Hunan from the end of October to mid‐March in 2023 and 2024, and from early February to mid‐May in 2024; in Gansu from mid‐May to end‐September in 2022; in Tianjin from mid‐April to mid‐July in 2021; and in Inner Mongolia from mid‐May to mid‐September in 2019.

In vitro seedlings were used for field trials in Guangzhou. Specifically, 14‐day‐old healthy potato plantlets were selected and transplanted into a plant growth chamber at 25 °C with a 10‐h light/8‐h dark cycle for 3 weeks. After this period, uniformly growing potato seedlings were transplanted to the field. In those regions outside Guangzhou, we used seed tubers produced from field‐grown plants derived from in vitro seedlings in Guangzhou. These selected seed tubers weighed between 100 and 150 grams to ensure their healthy status and uniformity.

All field experiments were conducted using a factorial randomized block design with 3 replicates. Two different planting methods were used in the diverse districts (Figure S12). In Guangzhou and Hunan, the planting density was 60 × 25 cm with a double‐row planting mode with a ridge height of 25 cm, width of 90 cm, and furrow width of 30 cm (Figure S12a). In Gansu, Tianjin, and Inner Mongolia, the planting density was 60 × 25 cm with a single‐row planting mode with a ridge height of 25 cm (Figure S12b). Regular field management, including irrigation, fertilization, and disease prevention, was performed according to Chinese standard agricultural practices for potato cultivation (GB/T 31753‐2015).

The yield per plant of transgenic lines in the field was investigated, and the number of plants per line was specified correspondingly in the figure legend. During harvest, the aboveground stems and leaves of each potato plant were removed, retaining only the tubers. The tubers were then individually weighed to determine the yield per plant. In Inner Mongolia, Tianjin, and Guangzhou (2020 and 2023), an equal number of plants were sampled from each plot and combined for yield assessment. In Guangzhou (2024), Hunan, and Gansu, all plants were harvested for yield assessment. Plants were randomly sampled from each plot and combined for the above‐ground biomass assessment. Based on these individual plant yield data, the mean and standard deviation were calculated. Student's *t*‐test was used to analyse the significant differences in the yield per plant between wild‐type (WT) and GOC transgenic potato plants.

### Gene/protein expression and enzyme activity assays

Total RNA was isolated from leaves using TRIzol reagent (Life Technologies) and treated with RNase‐free DNase I (Amersham). cDNA was generated from the extracted RNA using SuperScript II Reverse Transcriptase (Vazyme, China). Gene expression was analysed using a Real‐Time PCR Detection System (Bio‐Rad, Hercules, CA), and the primers used are listed in Table S4. Proteins were extracted as described previously (Shen *et al*., 2019). Fresh leaves (0.2 g) were homogenized in 1 mL phosphate buffer (0.1 M, pH 8.0). The extract was centrifuged at 12 000 rpm for 20 min at 4 °C, and the supernatant was used for subsequent SDS‐PAGE and western blot analyses. *Os*GLO3 and *Ec*CAT were detected using a monoclonal anti‐His antibody (Abmart), and *Os*OxO3 was detected using an anti‐*Os*OxO3 rabbit polyclonal antibody (Shen *et al*., 2019). GLO activity was determined according to Li *et al*. (2021) with a few modifications (Li *et al*., 2021; Xu *et al*., 2009); CAT activity was measured by following the consumption of H_2_O_2_ (extinction coefficient of 43.6 M^−1^ cm^−1^) at 240 nm for 1 min (Zhang *et al*., 2015); and OxO activity was determined by a CN‐PAGE method according to Zhang *et al*. (2015) with some modifications. Fresh leaves (0.1 g) were ground in liquid nitrogen and homogenized in 400 μL Tris–HCl buffer (0.1 M, pH 8.0). The extract was centrifuged at 12 000 rpm for 10 min at 4 °C, and an appropriate supernatant was used for CN‐PAGE (Zhang *et al*., 2015).

### Gas exchange analysis

Gas exchange parameters were measured on the fourth fully expanded leaf (from the top of the potato plants at the tuber swelling stage) using an LI‐6800 system (LI‐COR). Diurnal curves of net photosynthetic rates were generated under natural air and light conditions in January. For the generation of the light‐response curves, the conditions were set as follows: leaf temperature at 25 °C, relative humidity at 60%, CO_2_ concentration of 400 μmol mol^−1^, and gradually increasing photon flux density from 0 to 2200 μmol m^−2^ s^−1^. For the generation of CO_2_‐response curves, the conditions were set as follows: leaf temperature at 25 °C, relative humidity at 60%, photon flux density at 1600 μmol m^−2^ s^−1^, and the concentration of CO_2_ was detected with the environmental concentration of 400 μmol mol^−1^, gradually decreased to 50 μmol mol^−1^, returned to the environmental concentration of 400 μmol mol^−1^, and gradually increased to the maximum concentration of 2000 μmol mol^−1^. The measured data were fitted for light‐ and CO_2_‐response curves by Ye *et al*. (2013) using the online *Photosynthesis Model Simulation Software* (http://photosynthetic.sinaapp.com/), through which the LS, *A*_max_, and maximum rate of *V*_cmax_ were estimated. The photorespiration rate was estimated by subtracting the value of Pn at 21% O_2_ from that at 2% O_2_, according to Yeo *et al*. (1994). The dark respiration rate (*R*d) and the photorespiratory CO_2_ compensation point (Γ*) were determined using the Laĭsk method (Laĭsk, 1977; Von Caemmerer, 2000).

### Analysis of amino acids in potato leaves

Extraction of free amino acids was based on a method reported previously with some modifications (Zhang *et al*., 2016). Fresh leaves were sampled at 0.2 g and homogenized in 1 mL of 6% 5‐sulphosalicylic acid immediately. After an ice bath for 60 min, the homogenate was centrifuged at 5000 rpm for 10 min at a temperature of 15 °C, and then the supernatant was used for amino acid analysis. Free amino acids were analysed by Amino‐acid Analyser (SYKAM S‐433D, German).

### Leaf area, biomass, and yield analysis

The leaf area and biomass analyses were performed as described by Donnelly *et al*. (2001). Briefly, five field‐grown potato plants were randomly selected, and their leaves were removed. Twenty‐five leaves were randomly selected, aligned by their veins, and two 1 cm^2^ holes were punched on each side. These leaf discs were dried in an oven at 75 °C to a constant weight and then weighed. The dry weight of 100 leaf discs (100 cm^2^) was recorded as *a* (in g), and the ratio of leaf area to dry weight was calculated as:

(1) $$\text{Ratio}=\frac{100}{a}\left({\mathrm{cm}}^{2}/g\right)$$

All the leaves of a potato plant were collected, and their dry weight was recorded as *b* (in g). Therefore, the leaf area per potato plant (cm^2^) was determined by:

(2) $$\text{Leaf Area}=\frac{100}{a}\times b\;\left({\mathrm{cm}}^{2}\right)$$

The aboveground tissues were oven‐dried at 105 °C for 30 min and dried at 75 °C to a constant weight before being weighed.

Tuber yield was determined based on a previously reported method with some modifications (Kim and Lee, 2019). Approximately 10–50 plants per sub‐block were randomly selected to measure the tuber yield. The tubers were harvested after uprooting the entire plant within a radius of approximately 20 cm and were collected by manual digging. The tubers were manually washed with water and air‐dried for approximately 1 h. The fresh weight of the tubers was weighed. Tubers with a fresh weight of less than 5 g were regarded as developing tuber initials and were excluded from the samples. The tuber yield of the potatoes was determined as the sum of the tubers' fresh weight per plant.

### Measurement of sucrose and starch contents

Leaves and stems were sampled between 14:00 and 16:00, and the samples were ground into powder in liquid nitrogen. The sample powder (0.5 g) was extracted three times with 80% ethanol at 80 °C for 30 min. After centrifugation at 5000 × ***g*** for 15 min, the supernatant was used to measure sucrose content, and the pellets were used to measure starch content. Sucrose content was measured according to the method described by Luo and Huang (2011), and starch content was measured according to the method described by Nakamura and Miyachi (1982).

## Funding

This work was supported by the National Key Research and Development Program of China (2020YFA0907600), the Major Program of Guangdong Basic and Applied Research (2019B030302006), and the National Natural Science Foundation of China (32070265).

## Author contributions

X. P. and X. L. conceived and designed the experiments; X. L., Y. L., Z. Y., B. S., M. L., X. Z., X. C., and X. L. performed the experiments; X. L., and Y. L. analysed the data; X. P., X. L., Y. L., and Z. Z. wrote the paper; X. P., Z. Z., and G. Z. revised and approved the final version of the paper.

## Conflict of interest

No conflict of interest is declared.

## Supporting information


**Figure S1** The core region of vector *pBIA13*.
**Figure S2** Structure of the chloroplast localization peptide PCS1.
**Figure S3** Yield of GOC potato planted in Guangzhou in 2020 and 2023.
**Figure S4** Yield and biomass of GOC potato planted in other regions of China.
**Figure S5** Yield of GOC potato under adverse conditions.
**Figure S6** Representative photographs of GOC potato plants grown in fields.
**Figure S7** The intercellular CO_2_ concentration in GOC potato plants.
**Figure S8** Leaf areas of GOC potato plants grown in Inner Mongolia.
**Figure S9** Tuber numbers of GOC potato plants grown in Guangzhou and Inner Mongolia.
**Figure S10** Stem diameter of GOC potato plants grown in Inner Mongolia and Guangzhou.
**Figure S11** Solar radiations in different areas during the growth period of potato.
**Figure S12** Schematic representation of field planting methods.
**Table S1** Gas exchange parameters and chlorophyll fluorescence in GOC potato plants.
**Table S2** Free amino acid content in leaves of WT and GOC transgenic plants.
**Table S3** Solar radiations in different areas during the growth period of potato.
**Table S4** Primers used for real‐time RT‐qPCR.

## Data Availability

The data that supports the findings of this study are available in the supplementary material of this article.
