# Supplementary material for: Synthetic photorespiratory bypass more stably increases potato yield per plant by improving photosynthesis
Source: Plant Biotechnol J. 2025 Apr 2;23(7):2526–36. doi: 10.1111/pbi.70076 (PMC12205882; doi:10.1111/pbi.70076)
Supplement: Supplementary file 1 — Figure S1 The core region of vector pBIA13. Figure S2 Structure of the chloroplast localization peptide PCS1. Figure S3 Yield of GOC potato planted in Guangzhou in 2020 and 2023. Figure S4 Yield and biomass of GOC potato planted in other regions of China. Figure S5 Yield of GOC potato under adverse conditions. Figure S6 Representative photographs of GOC potato plants grown in fields. Figure S7 The intercellular CO2 concentration in GOC potato plants. Figure S8 Leaf areas of GOC potato plants grown in Inner Mongolia. Figure S9 Tuber numbers of GOC potato plants grown in Guangzhou and Inner Mongolia. Figure S10 Stem diameter of GOC potato plants grown in Inner Mongolia and Guangzhou. Figure S11 Solar radiations in different areas during the growth period of potato. Figure S12 Schematic representation of field planting methods. Table S1 Gas exchange parameters and chlorophyll fluorescence in GOC potato plants. Table S2 Free amino acid content in leaves of WT and GOC transgenic plants. Table S3 Solar radiations in different areas during the growth period of potato. Table S4 Primers used for real‐time RT‐qPCR. [file PBI-23-2526-s001.docx]

**Synthetic photorespiratory bypass more stably increases potato yield per plant via improving photosynthesis**

Xiuling Lin ^1, 2, †^, Yuming Long^1, †^, Zhen Yao^1, 3^, Boran Shen^1^, Min Lin ^1^, Xiaofen Zhong ^1^, Xiaohong Chen^1^, Xiangyang Li^1^, Guohui Zhu ^1^, Zhisheng Zhang^1^*, Xinxiang Peng^1^*

Correspondence to: Zhisheng Zhang (zzsheng@scau.edu.cn), Xinxiang Peng (xpeng@scau.edu.cn)

**Supplementary Materials:**

Figure S1-S12

Table S1-S4

#### Supplemental figures


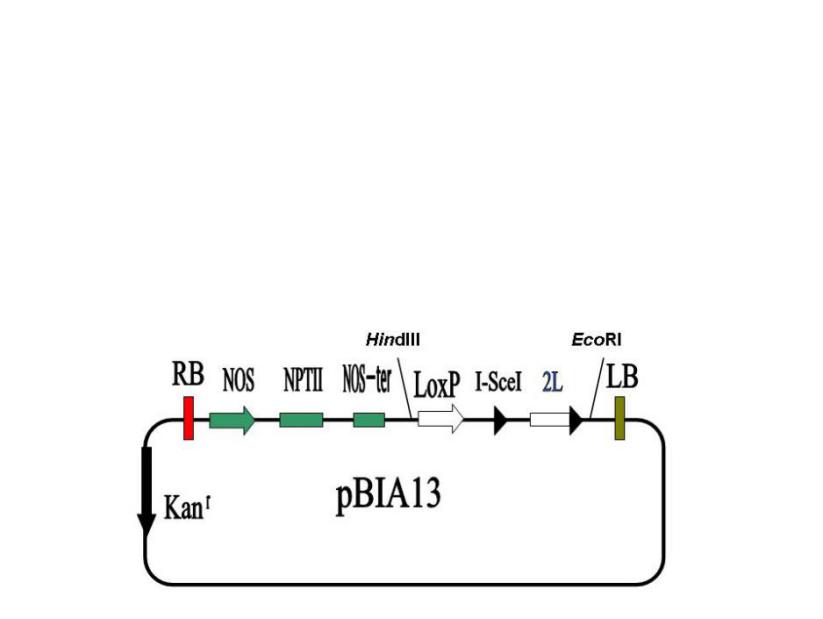


**Figure S1 The core region of vector *pBIA13*.**

Cre-loxP site-specific recombination (in dotted box):


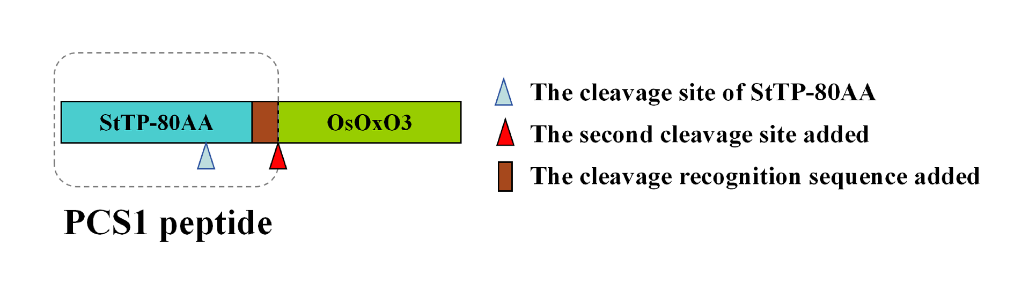
GTAACTATAACGGTCCTAAGGTAGCGAAGGATCCTGGCAAACAGCTATTATGGGTGTCGACCTGCAGTCATAACTTCGTATAGCATACATTATACGAAGTTATGGGCCGCATTACCCTGTTATCCCTAGGCCGCATAACTTCGTATAGCCTACATTATAGGATGGAGGGATATCCTCTCTTAAGGTAGC

**Figure S2 Structure of the chloroplast localization peptide PCS1.**

PCS1 (in dotted box) is constructed by adding 16 amino acids to the C-terminal of potato rbcS chloroplast localization peptide StTP-80AA. The cleavage recognition sequence with the 16 amino acids is adopted from the chloroplast localization signal peptide of plastocyanin precursor in *Silene pratensis*.

ATGGCTTCCTCTGTTATTTCCTCTGCAGCTGTTGCTACACGCACCAATGTTACACAAGCTGGCAGCATGATTGCACCTTTCACTGGTCTCAAATCTGCTGCTACTTTCCCTGTTTCAAGGAAGCAAAACCTTGACATCACTTCCATTGCTAGCAATGGTGGAAGAGTTAGGTGCATGCAGGTATGGCCACCAATTAACATGAAGAAGTACGAGACACTCTCATACCTTCCTGATTTGACTATGAGCTCAACCGTCAAGGTCGCCGTCGCCACCCCCAGGATGTCAATCAAGGCCTCCATG


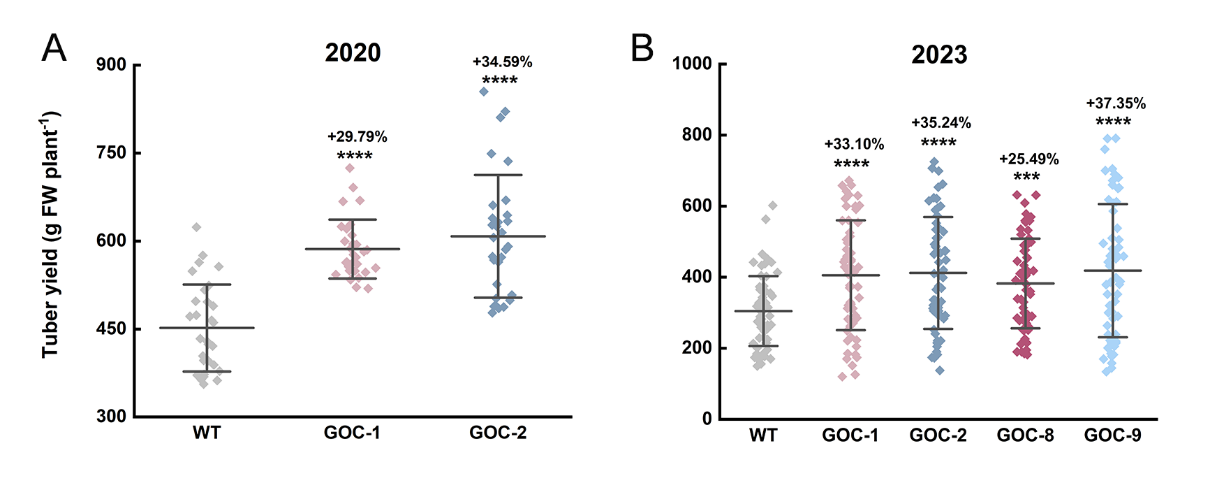


**Figure S3 Yield of GOC potato planted in** **Guangzhou in 2020 and 2023.**

**(A)** Yield per plant of GOC potato in 2020, n=30. **(B)** 2023, n=60. Data are presented as the mean ± SD; ****P* < 0.001, *****P* < 0.0001 according to Student’s *t*-test.


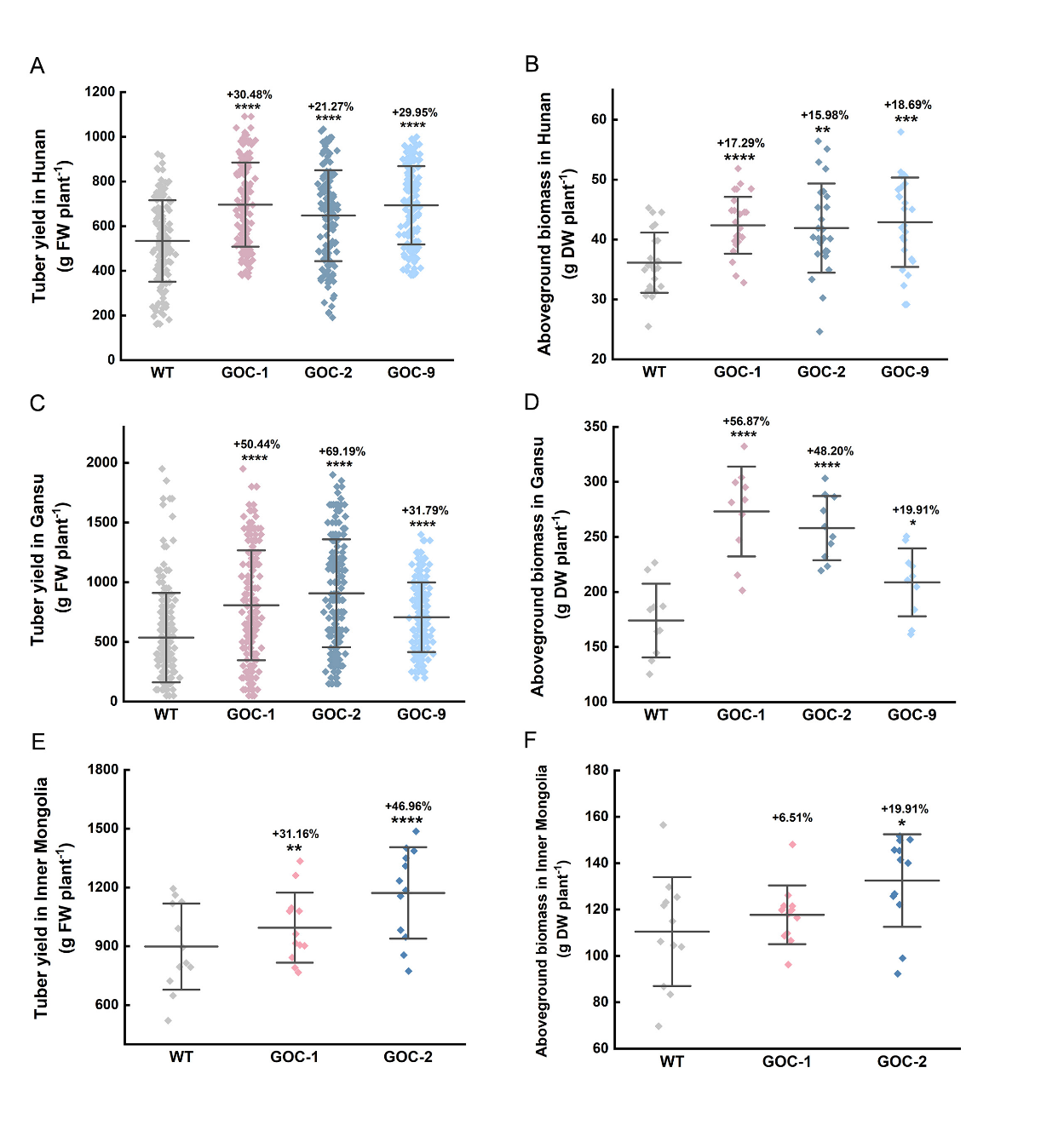


**Figure S4 Yield and biomass of GOC potato planted in other regions of China.**

**(A)** Yield per plant of GOC potato planted in Hunan in 2024, n=142. **(B)** Biomass of GOC potato planted in Hunan, n=26. **(C)** Yield per plant of GOC potato planted in Gansu in 2023, n=190. **(D)** Biomass of GOC potato planted in Gansu, n=10. **(E)** Yield per plant of GOC potato planted in Inner Mongolia in 2019, n=30. **(F)** Biomass of GOC potato planted in Inner Mongolia, n=12. Data are presented as the mean ± SD; **P* < 0.05, ***P* < 0.01, ****P* < 0.001, *****P* < 0.0001 according to Student’s *t*-test.


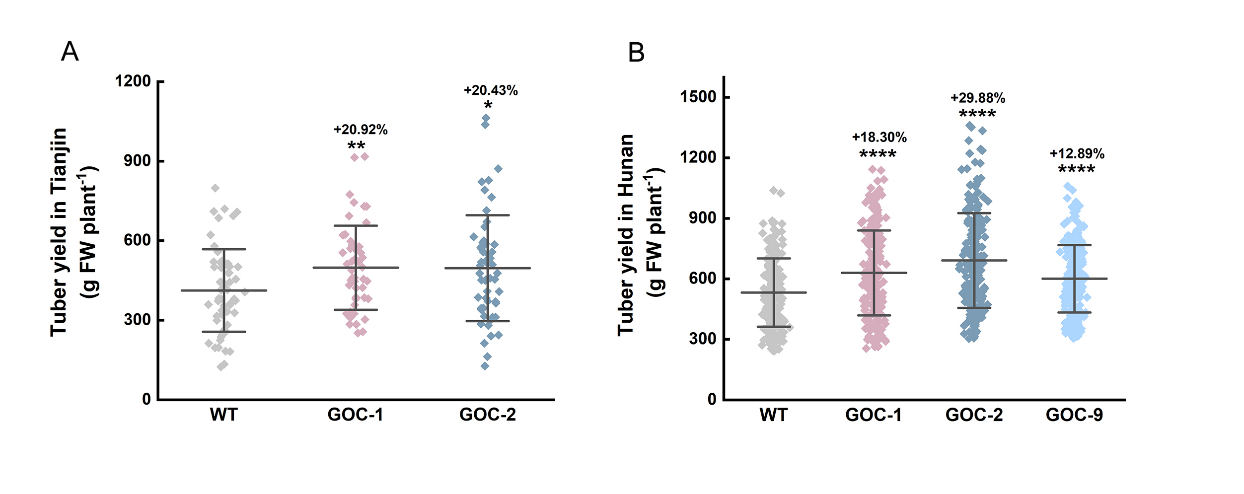


**Figure S5 Yield of GOC potato under adverse conditions.**

**(A)** Yield per plant of GOC potato planted in Tianjin (water logging condition) in 2021, *n=*52. **(B)** Yield per plant of GOC potato planted in Hunan in 2024 (low light and rainy conditions), *n=*228. Data are presented as the mean ± SD; **P* < 0.05, ***P* < 0.01, *****P* < 0.0001 according to Student’s *t*-test.

**
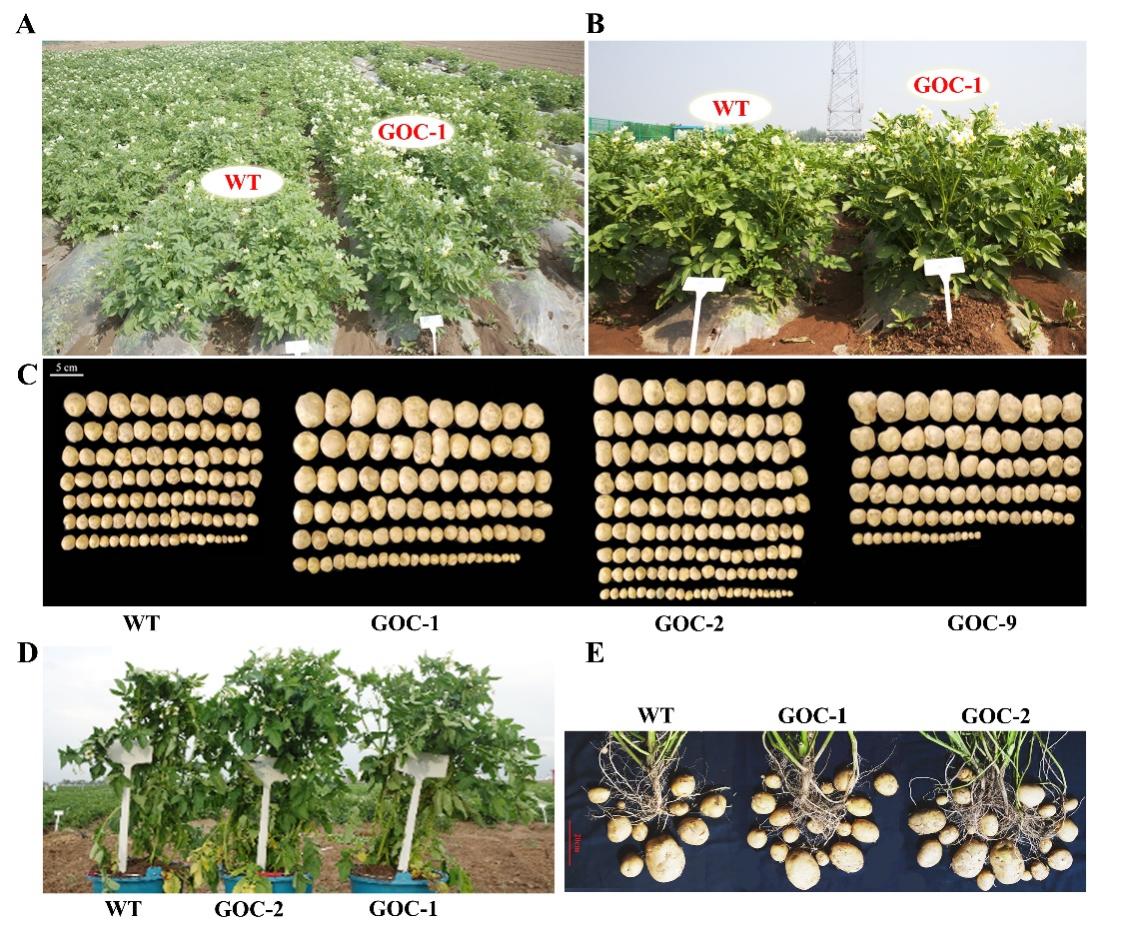
**

**Figure S6 Representative photographs of GOC potato plants** **grown in the fields.**

**(A-B)** Photographs of potato plants at booting stage grown in the field in Gansu. **(C)** Photographs of potato tubers harvested from 10 potato plants of WT and GOC grown in the field in Gansu. **(D)** Photographs of potato plants at harvest stage grown in the field in Inner Mongolia, which were transferred to pots for taking picture. **(E)** Photographs of potato tubers harvested from a representative potato plant of WT and GOC grown in Inner Mongolia.


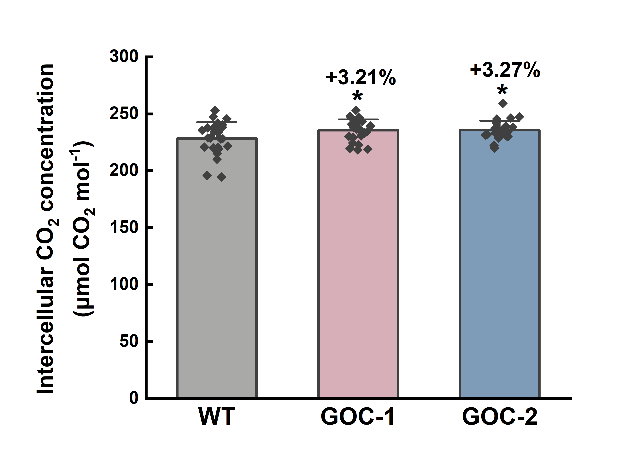


**Figure S7 The intercellular CO_2_ concentrations in GOC potato plants.**

Plants were grown in pots containing loose soil under greenhouse conditions. The fourth fully expanded leaf from the top of potato at tuber swelling stage was used for the determination, the conditions were set as follows: leaf temperature at 25℃, CO_2_ concentration is about 400 μmol mol^-1^, and photon flux density is 1600 μmol m^-2^ s^-1^. Data are presented as the mean ± SD, *n=*30; **P* < 0.05, according to Student’s *t*-test.


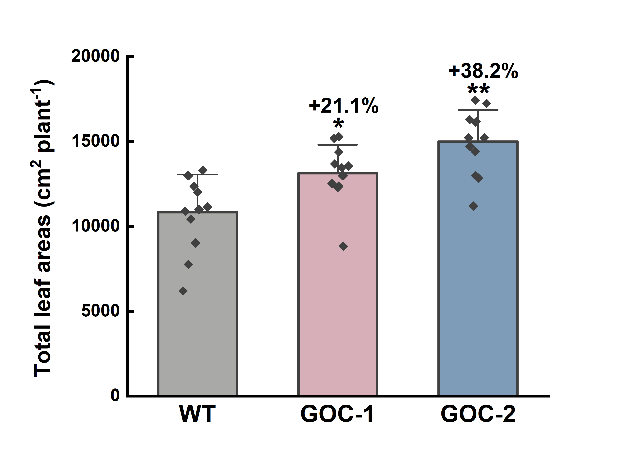


**Figure S8 Leaf areas of GOC potato plants grown in Inner Mongolia.**

Leaf areas were determined at tuber swelling stage (*n=*12). Data are presented as the mean ± SD; **P* < 0.05, ***P* < 0.01, according to Student’s *t*-test.


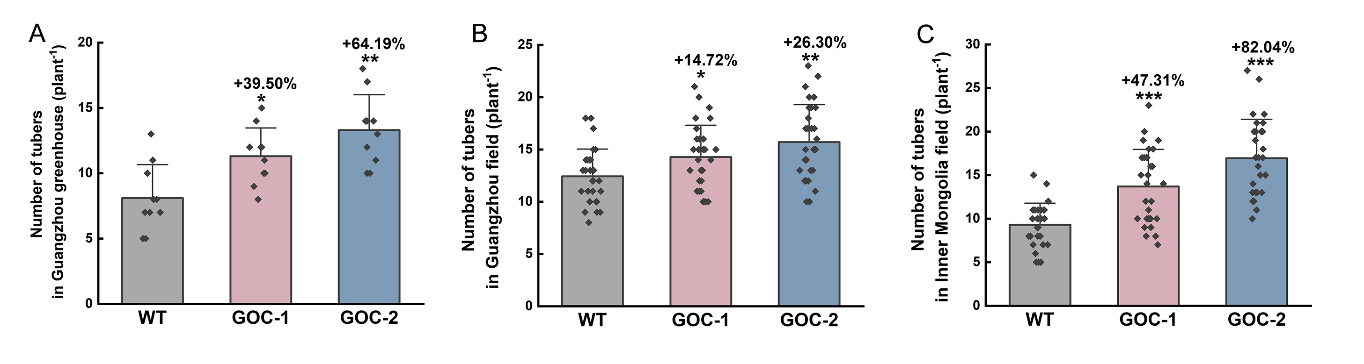


**Figure S9 Tuber numbers of GOC potato plants grown** **in Guangzhou and Inner Mongolia**

**(A)** Tuber numbers per plant grown in Guangzhou greenhouse (*n=*10). **(B)** Tuber numbers per plant grown in Guangzhou field (*n=*30). **(C)** Tuber numbers per plant grown in Inner Mongolia field (*n=*30). Tuber numbers were determined at harvest stage. Data are presented as the mean ± SD; **P* < 0.05, ***P* < 0.01, ****P* < 0.001, according to Student’s *t*-test.


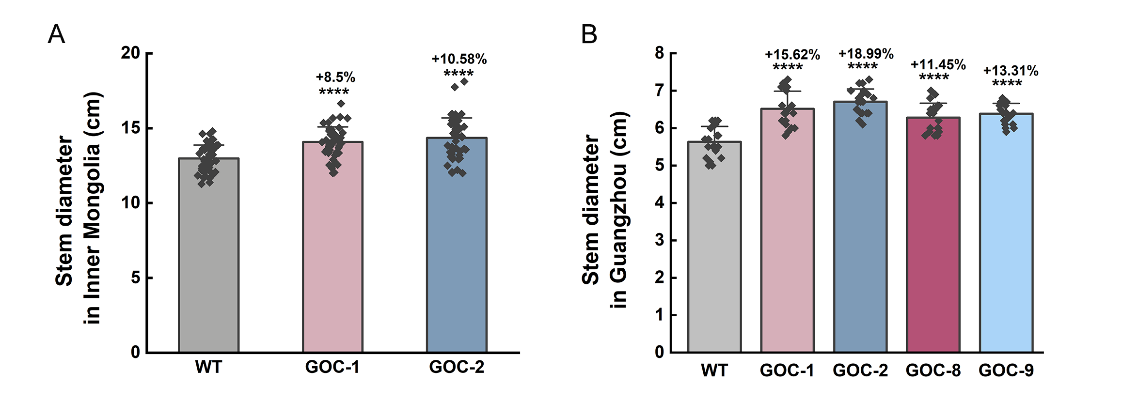


**Figure S10 Stem diameters of GOC potato plants grown in Inner Mongolia and Guangzhou.**

**(A-B)** Stem diameter was determined at tuber swelling stage in Inner Mongolia (*n=*50) and in Guangzhou (*n=*20). Data are presented as the mean ± SD; *****P* < 0.0001, according to Student’s *t*-test.

**
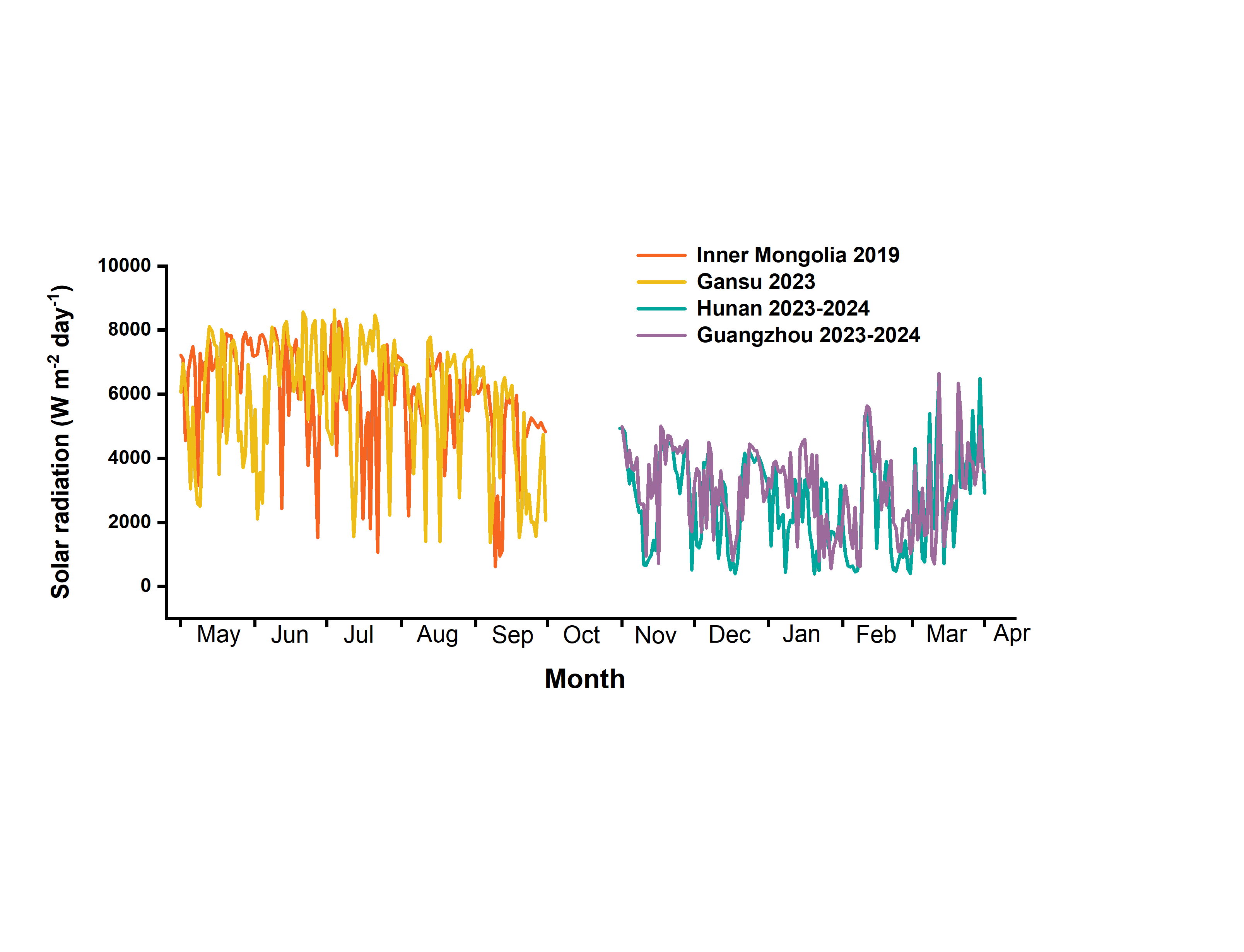
**

**Figure S11** **Solar radiations in different areas during the growth period of potato.**

The figure showed the daily total solar radiation in Gansu and Inner Mongolia from May to September in 2019 and 2023, and the daily total solar radiation in Guangzhou and Hunan from November 2023 to March 2024. Data are based on historical reanalysis datasets from the European Centre for Medium-Range Weather Forecasts (ECMWF)/National Aeronautics and Space Administration（NASA), provided by [www.xihe-energy.com](http://www.xihe-energy.com).


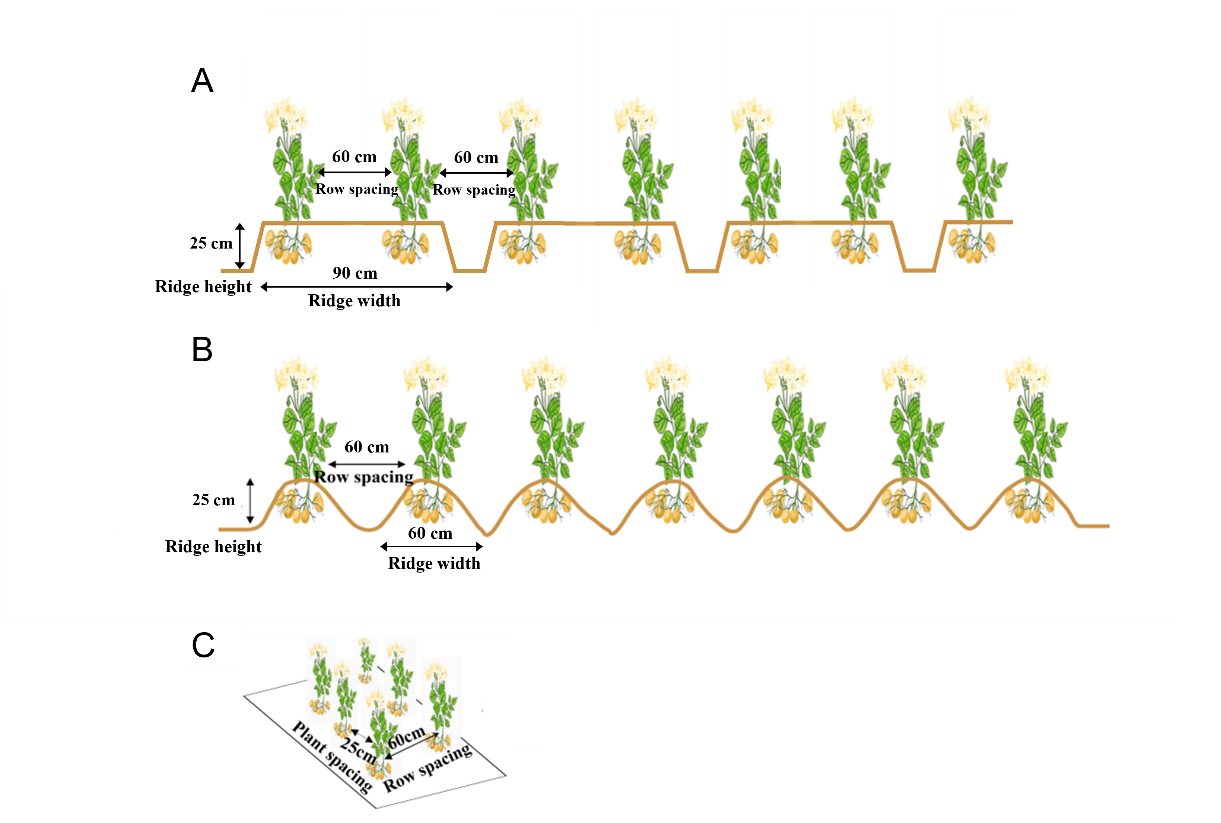


**Figure S12 Schematic representation of field planting methods.**

**(A)** Schematic representation of field planting methods of Guangzhou and Hunan. The ridge height is about 25 cm, the width is about 90 cm, and the furrow width is about 30 cm. **(B)** Schematic representation of field planting methods of Tianjin, Gansu and Inner Mongolia regions. The ridge height is about 25 cm, the width is about 60 cm. **(C)** Schematic top view of field planting. The planting density of the plants was set with a row spacing of 60 cm and a plant spacing of 25 cm within each row.

#### Supplemental tables

**Table S1** **Gas exchange parameters and chlorophyll fluorescence in GOC potato plants.**

|  | WT | | GOC-1 | | GOC-2 | |
| --- | --- | --- | --- | --- | --- | --- |
| Light saturation point (LSP, μmol m^-2^ s^-1^) | | 1730±22 | | 1841±61* | | 1887±93* |
| Dark respiration rate (*R*d, μmol CO_2_ m^-2^ s^-1^) | | 0.349±0.038 | | 0.490±0.038** | | 0.494±0.015** |
| Light-saturated photosynthetic rate  (*A*_max_, μmol CO_2_ m^-2^ s^-1^) | | 23.53±1.50 | | 24.94±0.88* | | 25.73±0.61* |
| Photorespiratory CO_2_ compensation point (Γ*, μmol) | | 42.63±1.01 | | 37.76±1.28* | | 37.43±2.33* |
| Maximum carboxylation rate (*V*_cmax_, μmol CO_2_ m^-2^s^-1^) | | 38.87±2.51 | | 44.68±2.30* | | 45.88±2.87* |

LSP, *A*_max_ and *V*_cmax_ were estimated by Ye *et al*. (2013) methods with an online model *Photosynthesis Model Simulation Software* (PMSS) (<http://photosynthetic.sinaapp.com/>). *R*d and Γ* were determined using Laĭsk method (Laĭsk, 1997; Von Caemmerer, 2000), *n=*6; Mean ± SD; **P* < 0.05, ***P* < 0.01 according to Student’s *t*-test.

**Table S2** **Free amino acid content in leaves of WT and GOC transgenic plants.**

| μg g^-1^ FW | WT | GOC-1 | GOC-2 |
| --- | --- | --- | --- |
| Phosphoserine (P-Ser) | 33.47±2.73 | 27.16 ±2.41* | 26.69 ±0.76** |
| Taurine (Tau) | 1.41 ±0.02 | 1.36 ±0.10 | 1.47 ±0.01 |
| Phosphoethanolamine (P-ser) | 7.43 ±0.66 | 6.61 ±0.58 | 7.41 ±0.48 |
| Aspartate (Asp) | 124.84 ±7.27 | 143.45 ±3.64** | 156.48 ±3.15*** |
| Threonine (Thr) | 35.02 ±2.02 | 49.28 ±1.21*** | 32.81 ±0.68 |
| Serine (Ser) | 65.10 ±3.79 | 65.64 ±1.40 | 48.58 ±1.18** |
| [Asparagine](javascript:;) (Asn) | 24.81 ±1.53 | 29.32 ±0.95** | 15.85 ±0.53*** |
| [Glutamic](javascript:;) [acid](javascript:;) (Glu) | 283.17 ±14.75 | 256.16 ±23.00 | 289.48 ±8.84* |
| Glutamine (Gln) | 635.74 ±35.90 | 584.39 ±25.54 | 498.27 ±14.03*** |
| Glycine (Gly) | 35.10 ±2.12 | 18.59 ±1.64*** | 10.59 ±0.31*** |
| Alanine (Ala) | 77.07 ±5.33 | 60.24 ±3.91*** | 39.13 ±0.84*** |
| Citrulline (Cit) | 22.86 ±1.52 | 27.45 ±3.00 | 16.32 ±2.51 |
| Valine (Val) | 17.22 ±0.93 | 12.33 ±0.54*** | 9.60 ±0.11*** |
| Methionine (Met) | 1.34 ±0.19 | 1.42 ±0.29 | 1.00 ±0.09 |
| Isoleucine (Ile) | 8.21 ±0.18 | 6.70 ±0.72* | 5.00 ±0.42*** |
| Leucine(Leu) | 3.53 ±0.19 | 2.78 ±0.70 | 2.22 ±0.46* |
| Tyrosine (Tyr) | 7.90 ±0.56 | 10.04 ±0.21*** | 8.21 ±0.24 |
| Phenylalanine (Phe) | 20.28 ±1.49 | 18.76 ±0.51*** | 17.32 ±0.87 |
| γ-aminobutyric acid (γ-ABA) | 189.75 ±17.82 | 198.07 ±34.34 | 148.98 ±7.72* |
| Histidine (His) | 1.16 ±0.24 | 1.83 ±0.23** | 1.97 ±0.06** |
| Ornithine (Orn) | 3.75 ±0.26 | 2.01 ±0.04*** | 1.78 ±0.06*** |
| Lysine (Lys) | 3.30 ±0.28 | 3.61 ±0.17 | 3.79 ±0.07* |
| Arginine (Arg) | 2.92 ±0.50 | 3.20 ±0.09 | 2.54 ±0.27 |
| Proline (Pro) | 6.52 ±0.70 | 9.87 ±0.53** | 8.25 ±0.18* |
| Urea (Urea) | undetected | 10.57±1.56 | 10.74±0.87 |
| Summation | 1611.9 | 1550.84 | 1364.68 |

Amino acids were extracted in the 4th fully expanded leaves from the top of potato plants at the tuber swelling stage, and then were determined by an Amino-acid Analyzer (SYKAM S-433D) (n=4, Mean ± SD; **P* < 0.05, ***P* < 0.01, ****P* < 0.001 according to Student’s *t*-test).

**Table S3** **Solar radiations in different areas during the growth period of potato.**

| Region | Province | Coordinates | | Potato planting to harvesting date | | Solar radiation per day (W m^-2^) | Total solar radiation  (W m^-2^) |
| --- | --- | --- | --- | --- | --- | --- | --- |
|  |  | Latitude | Longitude |  |  |  |  |
| South China | Guangzhou | 23˚07′53"N | 113˚15′36"E | | Mid-November to Mid-March | 3046 | 462969 |
| South-Central China | Hunan | 25˚46′24"N | 113˚00′32"E | | End-October to Mid-March | 2883 | 616871 |
| Northwest China | Gansu | 36˚03′42"N | 103˚49′54"E | | Mid-May to End-September | 5806 | 888370 |
| North China | Inner Mongolia | 40˚50′25"N | 111˚44′35"E | | Mid-May to Mid-September | 5982 | 915218 |

Data are based on historical reanalysis datasets from the European Centre for Medium-Range Weather Forecasts (ECMWF)/National Aeronautics and Space Administration（NASA), provided by [www.xihe-energy.com](http://www.xihe-energy.com).

**Table S4 Primers used for real-time RT-qPCR.**

| Gene | Primer sequence |
| --- | --- |
| *OsGLO*3 | 5’- GGCCGGTATCATCGTGTCCAAC -3’  5’- GCCTCCCTGACGACCTCTTCC -3’ |
| *EcCAT* | 5’- CGGCTTTCCATCCTGGGCATATC -3’  5’- GGTCGGACGGTTAATCGGAATCTC -3’ |
| *OsOxO3* | 5’- GGTGGTGTCCTTCAACAGCC -3’  5’- AACTACACCAGCATCCACGC -3’ |
| *APT* | 5’- GAACCGGAGCAGGTGAAGAA -3’  5’- GAAGCAATCCCAGCGATACG-3’ |
